# Ground Truth Data Generator for Eye Location on Infrared Driver Recordings

**DOI:** 10.3390/jimaging7090162

**Published:** 2021-08-27

**Authors:** Sorin Valcan, Mihail Gaianu

**Affiliations:** 1 Department of Computer Science, West University of Timişoara, 300223 Timişoara, Romania; mihail.gaianu@e-uvt.ro; 2 Continental Automotive Romania VNI HMI, 300704 Timişoara, Romania

**Keywords:** labeling automation, infrared camera, driver monitoring, eye detection, neural networks

## Abstract

Labeling is a very costly and time consuming process that aims to generate datasets for training neural networks in several functionalities and projects. In the automotive field of driver monitoring it has a huge impact, where much of the budget is used for image labeling. This paper presents an algorithm that will be used for generating ground truth data for 2D eye location in infrared images of drivers. The algorithm is implemented with many detection restrictions, which makes it very accurate but not necessarily very constant. The resulting dataset shall not be modified by any human factor and will be used to train neural networks, which we expect to have a very good accuracy and a much better consistency for eye detection than the initial algorithm. This paper proves that we can automatically generate very good quality ground truth data for training neural networks, which is still an open topic in the automotive industry.

## 1. Introduction

Training neural networks requires large datasets with good accuracy in order to have a general and precise detection model. The generation of these datasets is a very expensive and time consuming aspect of this process, with whole teams of people working in data marking. Of course, there are specific tasks, such as price prediction or sentiment analysis, where data already exist on the internet because they are naturally generated by people. For the problem addressed in this paper, which is eye location in infrared images of drivers, this marked data can be very difficult to obtain.

Another big problem in the labeling process is that hours of marking eyes on images is a very strenuous task and it always leads to natural human errors. This usually leads to a double checking process for the labeling, which means additional time and effort and it does not guarantee the expected quality improvement.

This paper presents an algorithm the main purpose of which is to generate datasets that contain images labeled for eyes location. Detection is carried out on grayscale images obtained from an infrared sensor with a resolution of one megapixel. This sensor is specific for the automotive industry since it offers good and similar visibility during day and night. The resolution may differ depending on the sensor generation. Implementing such an algorithm has many advantages such as a huge time improvement in dataset generation and the removal of human errors.

We used data generated in real conditions but there are also techniques for generating synthetic data, which are described in [1].

The most common procedure for collecting ground truth data is where humans manually annotate. This procedure is used in tasks such as the pupil counter ellipse when evaluating pupil detection algorithms, as in [2,3].

Various eye image datasets generated by eye trackers already exist [4,5,6,7,8,9,10], including recordings from driver studies as well as simulators [4,5,6,7]. In addition, there are also recordings from specific challenges [10] as well as real time capable algorithms [11,12,13,14,15].

There are entropy-based methods for eyes detection, such as in [16,17], which show good accuracy on public datasets with grayscale images. However, those images have different properties compared to the ones obtained from the automotive infrared sensor. In our dataset, the face is strongly highlighted and eye components, such as pupil, iris and sclera, have different intensities. Our algorithm is developed for these specific properties of the image.

## 2. Methods

The proposed method for detecting eyes on driver faces is based on converting the grayscale input picture and possible eye patches to only three possible values for pixels: black, gray or white. This is based on two thresholds which are calculated dynamically for each specific picture and area. Using the converted data we can identify specific proprieties of the patches that contain eyes.

Every face in the world is different. Even two images with the same face are different because of position and brightness. This method is novel and very efficient since it reduces all the noise and specific features of a face to a trivial three-color image but still has enough information to detect eyes with very good accuracy.

The grayscale pictures obtained from the infrared sensor, which is directed at the driver, offer very good visibility during both day and night. Even more importantly, the face of the driver is very well highlighted while the ceiling and the seat of the car are very dark. This helps us to focus only on the face area and not have to deal with many additional shapes around the face.

All the functionalities are implemented to make the algorithm independent from the input picture resolution. Therefore, during the detection process there are many parameters, the values of which are set in relation to the width and height of the image. The exact resolution of the frames used to develop the algorithm is exactly 1280 × 800. For future work, if there are recordings made with other sensors that have a higher resolution, nothing needs to be changed. The only thing that may differ is the car setup, which may require adapting the searching parameters for the new driver position in the car with respect to the field of view of the camera.

### 2.1. Threshold Computation

The algorithm for thresholds computation, which is used to convert a picture, is implemented to return three values: one threshold value which can be used to convert from grayscale to black and white and two others that are used to convert from grayscale to black–gray–white.

**Figure 1:**
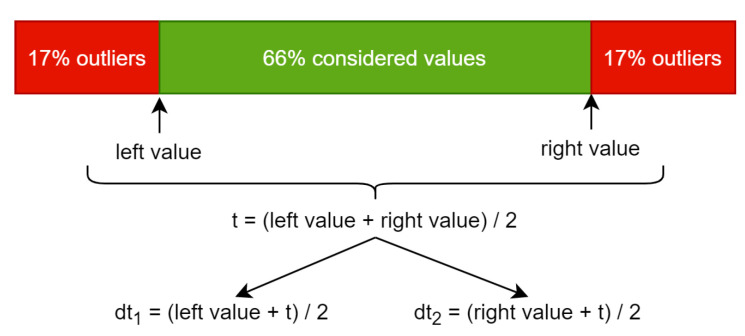
The process of computing thresholds. t represents the threshold which can be used to convert grayscale to black and white. dt_1_ and dt_2_ represents the thresholds used to convert from grayscale to black–gray–white.

In order to obtain them we perform computations using a histogram. A value of a pixel is stored on one byte so the histogram will have a length of 256. The value at a specific index represents the counter of appearances of that pixel value in the given image. The left value in Figure 1 represents the index from the histogram, which we reach after adding the counters from left to right until touching or exceeding 17% of the total pixel count. The right value is similar, but the counting is done from right to left. Using the left value and the right value we compute the average and this is the threshold (t) that can be used to convert the input image to black and white.

Because we are interested in more details than the black and white converted image can provide, this threshold is split into dt_1_ and dt_2_, which stand for double thresholds 1 and 2. This is done by computing the average between t and the left value to obtain dt_1_ and the same using the right value for dt_2_. Using these two resulting thresholds, we can convert the grayscale image to black–gray–white using the following function, which is applied for every pixel:

(1) $$p(x)\;=\;\left\{\begin{array}{cc} 0 & x\;<\;{\mathrm{dt}}_{1}\\ 127 & {\mathrm{dt}}_{1}\leq x\leq {\mathrm{dt}}_{2}\\ 255 & x\;>\;{\mathrm{dt}}_{2}. \end{array}\right.$$

**Figure 2:**
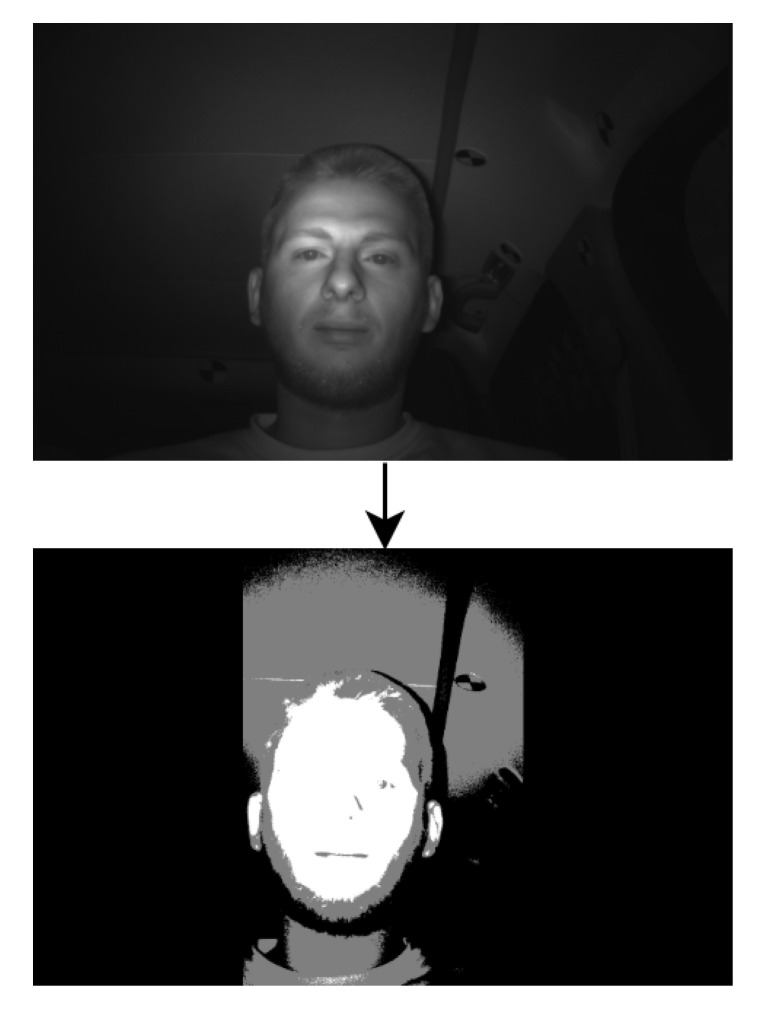
Example of input image converted from grayscale to black–gray–white.

The procedure for cutting edges to remove outliers is performed for safety in the case of extreme values appearing in overexposed frames. This percentage of outliers’ removal is configurable but the resulting threshold was similar for values between 5% and 20% in many cases, with a difference up to four units. However, there is no definition of a perfect threshold for a specific frame. The most important thing is to have a resulting image in which specific features of the face can be observed. Figure 2 presents an example of a converted image using the two thresholds.

### 2.2. Face Area Selection

The selection of the face area has the purpose of providing a bounding box that describes the four boundaries where the face is located: left, right, up and down. This bounding box will be used by the eye selection algorithm to search for the eyes.

Figure 2 describes the first step in the process. It can be observed that on the left and right of the converted image, the whole area is black. The thresholds are computed and applied only in the following bounding box:

- left limit is 30% of picture width;
- right limit is 70% of picture width;
- up limit is 0;
- down limit is picture height −1.

Please note that the coordinate system has the origin in the top left corner of the 2D image, with the x axis positive to the right and the y axis positive downward.

This means the face area will not be selected if the head is not located between these boundaries. The eye selection algorithm uses its own tracking system based on the previous eye location, so this face area is only important for the initial localisation.

**Figure 3:**
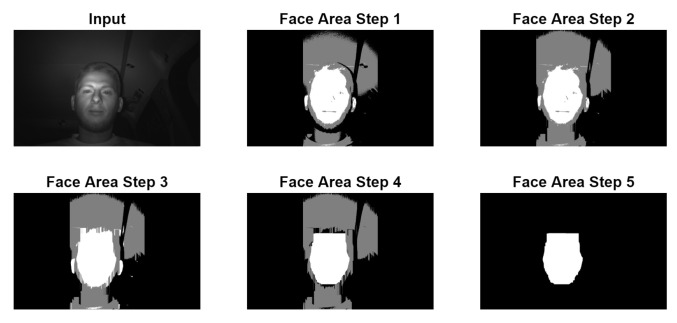
Example of input image converted from grayscale to black–gray–white.

The converted picture goes through more cleanup stages before the computation of the final bounding box as shown in Figure 3:

- Face Area Step 1 represents the application of the double threshold to the input image;
- Face Area Step 2 is the replacement of all vertical black lines which have a size of less than 6.5% of the picture height with white. This is done to remove any black horizontal line that may cut the white head area into two pieces, which may be caused by the driver wearing glasses;
- Face Area Step 3 represents the replacement of gray lines with white lines, similar to step 2;
- Face Area Step 4 is the replacement of all horizontal white lines which have a size of less than 7.8% of the picture width with black. This is done to remove the white noises in the upper and lower side of the white area which may be caused by hair. This noise can lead to a bounding box starting much higher than the actual face location;
- Face Area Step 5 is the actual selection of the big white area representing the face. This is performed with a recursive algorithm that goes through all the white pixels. The starting point for the recursive part is represented by the middle of the thresholded area. During this recursive process, the four limits of the bounding boxes are set. In case the starting point does not land exactly on a white pixel, the starting point is moved up and down until a white pixel is found in one, both or none of the two directions. When a white pixel is found in both directions, two possible face areas are computed and the one with smallest ratio between the width and the height of the bounding box is kept.

The result of the face area selection is represented by a bounding box defined by four values:

- lx: x coordinate of the left most white pixel in the face area;
- rx: x coordinate of the right most white pixel in the face area;
- ty: y coordinate of the up most white pixel in the face area;
- by: y coordinate of the lowest white pixel in the face area.

### 2.3. Eye Selection

The eye selection algorithm searches in the face area for a pair of eyes. This means that it will output the eye location only if both of them are detected together as a pair. In order to detect them, both eyes need to be open. The output is defined by two bounding boxes for the left and right eyes, which both contain the eye, even if the pupil is not exactly in the middle of the bounding box. One bounding box is defined by the following parameters:

- ltx: left top x coordinate in pixel;
- lty: left top y coordinate in pixel;
- w: width of the bounding box;
- h: height of the bounding box.

It should be noted that the bounding box for the left eye represents the location of the person’s left eye. This means that the bounding box for the left eye will have a greater x coordinate value than the one for the right eye.

First we need to set the area in which the eyes will be searched for. There are two ways in which this area can be set: using the eye locations which were found on the previous frame in the case of a successful detection, or otherwise using the bounding box which was computed by the face area selection algorithm on the current frame. The area where the eyes will be searched for is similar to the bounding box used for the face area.

When the eye locations from the previous frame are available, the bounding box will have a width of 26% of the picture’s width and a height of 23% of the picture’s height. The way the bounding box is computed is as follows:

- compute the average x and y coordinate of the left and right eye location from previous frame using:
  (2) avgY=(leftEyeTopY+rightEyeBottonY)/2
  (3) avgX=(rightEyeLeftX+leftEyeRightX)/2
- using the average values compute the eye search area bounding box:
  (4) lx=avgX−(bbw/2)
  (5) rx=avgX+(bbw/2)
  (6) ty=avgY−(bbh/2)
  (7) by=avgY+(bbh/2)
  where bbw and bbh are the dimensions of the search area bounding box mentioned before.

In the case when the eyes were not detected in the previous frame, then the bounding box for the eye searching area will be defined using the face area. This is done by taking lx from the face area and adding to it 35% of the picture’s width to compute rx. In the same way, we take ty and add to it 37% of the picture’s height in order to have by. This resulting bounding box is bigger in both width and height than the one computed using the eyes from the previous frame, because in that case we are sure that the eyes will not move very much from one frame to another so we trust a smaller area in which to search for the eyes again. It makes the algorithm more efficient when this tracking process is used. When using only the face area, we use a bigger window to increase the chances of finding the eyes when encountering noisier data because of hair.

Once the bounding box for the eye search is defined, this area is traversed by a window, which has a width 5.8% of the picture’s width and a height of 8.7% of the picture’s height using a stride of 1.1% of the picture’s width in the x direction and 1.2% of the picture’s height in the y direction. This size was chosen for the window because all the eyes in the recordings used to test the algorithm would fit inside. By traversing the eye search area with this window we obtain multiple possible eye patches. Two of these eye patches will be selected in the end as eyes and their location will be used to fill the output of the algorithm defined above as two bounding boxes. The output of the eye bounding boxes will always have a fixed width and height which is actually the width and height of the traversing window for obtaining the possible eye patches.

**Figure 4:**
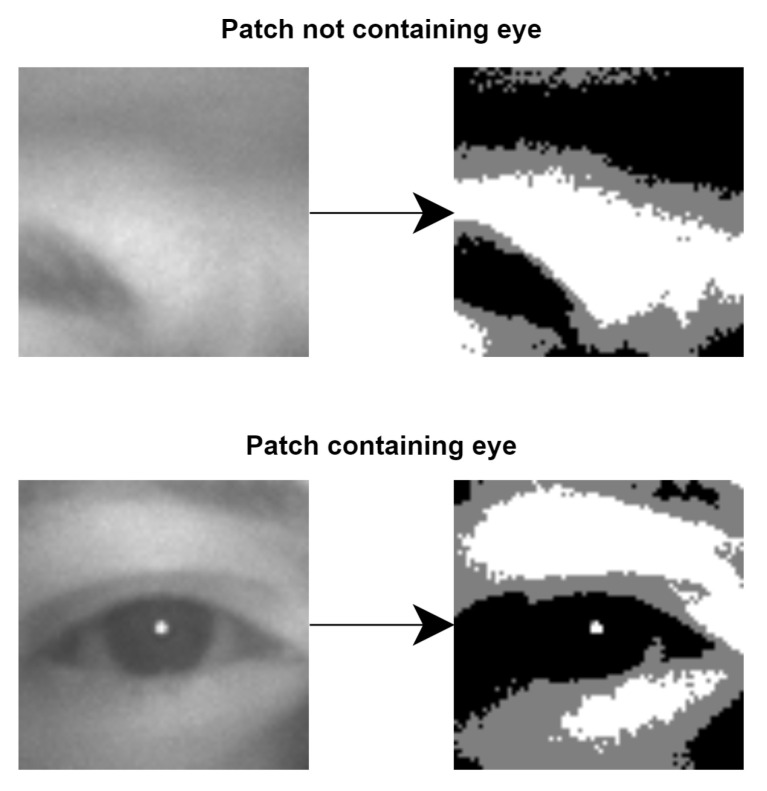
Top is a patch from the search area which does not contain an eye converted from grayscale to black–gray–white. Bottom is a patch from the search area which definitely contains an eye converted from grayscale to black–gray–white.

After all possible eye patches are selected from the searching window, all of them are converted from grayscale to black–gray–white using the double threshold. Figure 4 shows two examples of converted patches and the level of detail we obtain.

Black–gray–white eye patches go through multiple selection steps until there remain only the possible eye pairs.

### 2.4. Black Percentages Rule

**Figure 5:**
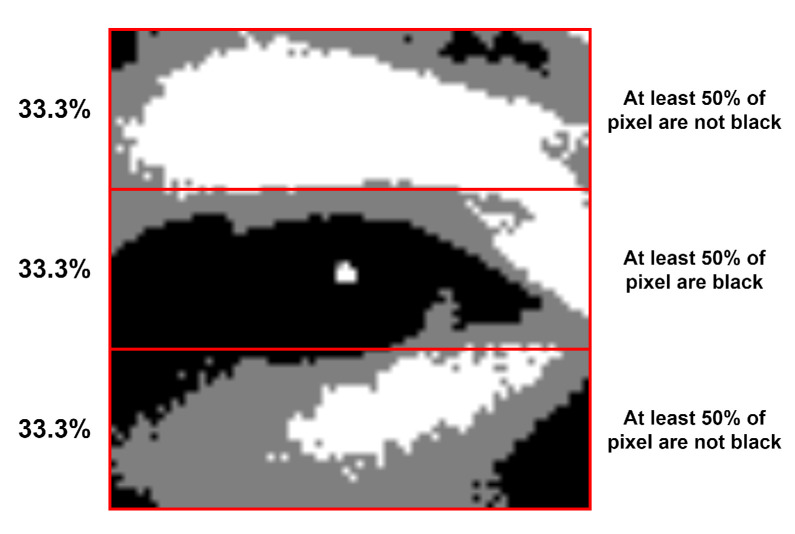
Black rule requirements for first patch’s selection step.

The first selection step is the black percentages rule, which will only keep those patches that meet the requirements described in Figure 5.

This rule removes more than 95% of the possible eye patches in almost every frame.

#### 2.4.1. Eye Ratio Map Score

**Figure 6:**
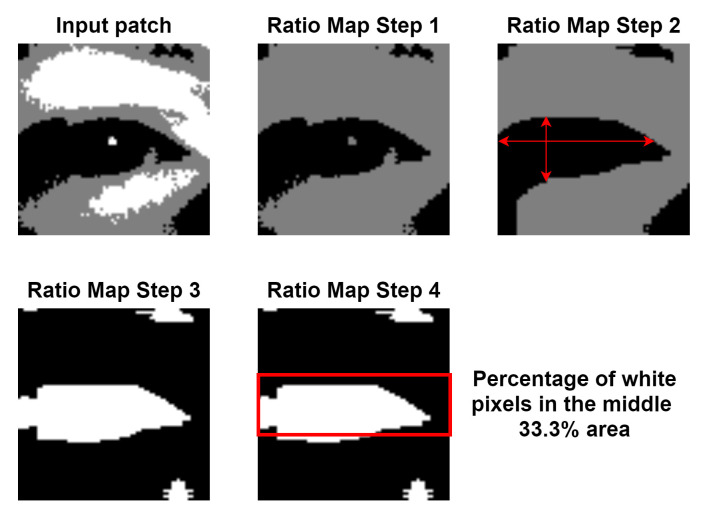
Visualisation of steps for eye ratio map score computation.

The eye ratio map score can be considered a confidence score that indicates how high the probability is that there is the shape of an eye inside the current patch. This score is not used to remove any of the remaining patches. There are four steps, which are visualised in Figure 6:

- Ratio Map Step 1 is the replacement of all white pixels in the current patch with gray pixels;
- Ratio Map Step 2 represents the computation of the ratio values. For each pixel in the patch we compute the size of the horizontal and vertical black lines of which the pixel is part. In case the current pixel is not black, both values will be 0. If the ratio between the horizontal and vertical size is greater than a predefined value of 1.3, then the pixel is replaced with white, otherwise it is set to black. The value of 1.3 is fixed and is used to check that the horizontal and vertical values computed for one pixel form the shape of a rectangle, which is approximately what the black area of the eye looks like;
- Ratio Map Step 3 represents the visualisation of the resulting ratio map from step 2;
- Ratio Map Step 4 is the actual computation of the score for the ratio map. The percentage of white pixels in the middle 33.3% area represents the final ratio map score for an eye patch.

#### 2.4.2. Pupil Reflection Check

Pupil reflection can be observed in the eye containing patch from Figure 4 and in Figure 5 as the small white area which is inside the 33.3% middle black area. For a possible eye patch, it is mandatory to contain it in order to keep it for the next selection step.

The pupil reflection check is carried out with a search inside the 33.3% middle area of the possible eye patch. All the white spots found in this area are represented by a bounding box with the same parameters as the face area and the total number of white pixels that are part of it. There are two major checks performed for each white spot in order for it to be considered as a pupil:

- the total number of white pixels forming the current spot must be greater than or equal to four and less than or equal to 24. Those values result from a general analysis of the majority of the pupil reflections on the recordings available for this dataset. However, for other recordings made with another sensor and a different infrared light setup, the pupil reflection was significantly different, and bigger values were necessary to detect the pupil. That is the reason those values are not relative to the picture width and height as are the majority of the parameters used in this algorithm;
- using the extreme points from the current bounding box, the spot is checked to be inside a black area. There must be at least three pixels in all four directions that are black. This is done because there may be other white spots that have the right dimension but may be located somewhere on the skin and this will not be considered a pupil.

The first white spot that passes both checks is considered to be a valid pupil and the possible eye patch is kept. Otherwise the patch is removed and will not be used for further computation.

**Figure 7:**
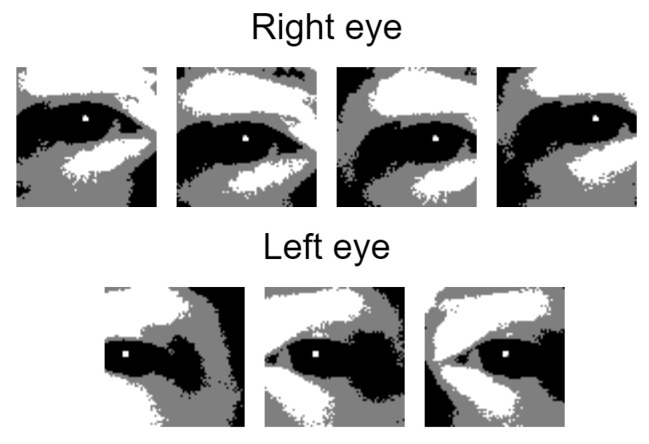
Remaining patches after the pupil reflection check is done.

This step results in a list of overlapping eye patches for both eyes, as described in Figure 7. There are very rare cases when the pupil check can be tricked by a very specific patch that is not actually an eye. Even if this happens, it is very difficult for it to be selected as a valid eye after the next steps are applied.

#### 2.4.3. Removal of Overlapping Patches

In most cases many of the remaining patches from the pupil reflection check are overlapping. Theoretically, all of them could be a valid result because all of them contain the eye inside. However, there must be a selection step that chooses which of them fits an eye the best.

To make this selection, a validation score is computed for each eye patch. This score will determine which patch is kept in a two by two duel of patches that overlap. The score is computed using the three black rule percentages from Section 2.4 and the ratio map percentage from Section 2.4.1 as follows:

(8) $$\mathrm{score}\;=\;b_{1}\;+\;b_{2}\;+\;b_{3}\;+\;r$$

where b_1_, b_2_ and b_3_ are the percentages from the black rule and r is the ratio map percentage.

In each duel, the patch with a smaller score is removed. The one that is kept is still available for duels with other patches until there are no overlapping ones remaining.

This does not mean there will be only two remaining patches after we remove the overlapping ones. There can be cases where we still have three, four or five patches remaining that do not overlap.

#### 2.4.4. Computing Possible Eye Pairs

Using the remaining patches, we use the x and y coordinates of the top left corner of every eye patch to determine whether two of them are valid to be considered an eye pair. This is done using some predefined mandatory distances between two patches:

(9) abs(y1−y2)≤maxY

(10) minX≤abs(x1−x2)≤maxX

where maxY is the maximum distance in the y direction between the two bounding boxes with a value that is 8.7% of the picture’s height; minX is the minimum distance in the x direction between the two bounding boxes with a value of 150% of the eye patch’s width; maxX is the maximum distance in the x direction between the two bounding boxes with a value of 300% of the eye patch’s width.

All pairs of eye patches that have distances between them fitted in these ranges will be valid for the final selection step.

#### 2.4.5. Selection of the Final Eye Pair

The best eye pair is selected from the overlapping patches using the score used in Section 2.4.3. This time the score of an eye pair is computed as the sum of the two patches’ scores. The pair with the best score is the one that will represent the output of the eye selection algorithm.

**Figure 8:**
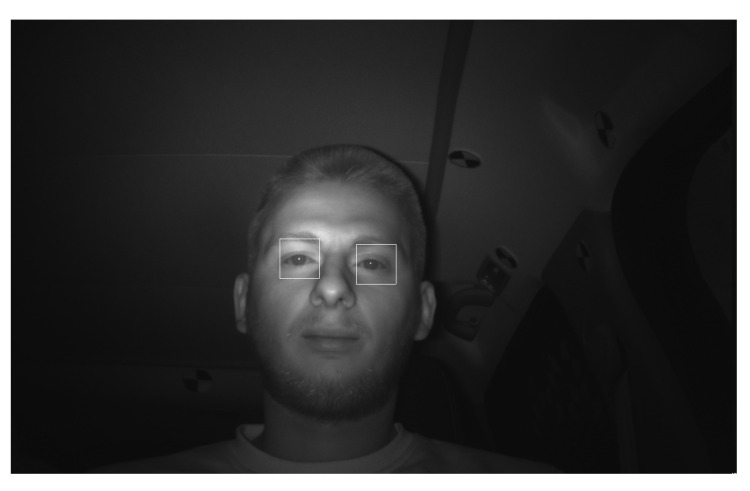
Result of the eye selection algorithm.

Because we use absolute values to compute possible eye pairs, as described in Section 2.4.4, at this point we do not know which is the left eye and which is the right eye. This requires the two patches to be ordered. The patch that has the greater x coordinate will be assigned to the left eye and the other one to the right eye. The output of the algorithm can be observed in Figure 8.

## 3. Obtained Results

The algorithm was run on 800 recordings with more than 100 different subjects. These recordings have different scenarios in which people are asked to look at specific markers on the board of the car, to get out of the car and back in, and real life driving situations. All these scenarios can be done at different times of the day, with different light situations depending on the weather.

Some subjects may appear in multiple recordings but in different outfits, such as wearing glasses, hats and/or surgical masks. They are from different ethnicities: Caucasian, African, Asian and Latino.

Using this set of recordings, there are approximately 2 million frames processed. The algorithm returned the eye location output on 397,906 frames.

In terms of precision, after a walk through the outputs the eyes were inside the computed bounding box in more than 95% of the cases. Most errors appear in frames where a white reflection from some specific glasses with black frames is small in size, which tricks the pupil check. This check is also responsible for many other frames where the eye may be detected, but the reflection does not meet the specified requirements in Section 2.4.2, especially when the head is rotated and the reflection is closer to the edge of the black area. However, the improvement of precision obtained with this check is much higher than the few frames which are wrongly detected because of the pupil reflection so the problem of glasses frames remains to be solved.

There are no frames where the algorithm computed an eye location when the driver was not present at the steering wheel. However, less than 0.1% of frames had wrong bounding boxes appear during the process of the driver getting behind the steering wheel. These cases are very difficult to treat at the moment.

This dataset may be automatically post processed in order to avoid continuous sections of almost identical frames as input for the neural network train. This will probably reduce the size by about half. It is part of the entire automatic system, where it is strictly forbidden to have any human input or correction over the dataset before the neural network training.

## 4. Future Work

The improvement of this algorithm goes in two main directions: improvements and optimisations of the current eye selection implementation and the addition of nostrils and mouth detection in order to have all the facial features available for the neural network input.

Having all these features of a face detected using this conversion of the grayscale image from the automotive infrared sensor to a three-color image can drastically reduce the costs in related projects and prove that we can reduce the problem of face feature extraction to a very small amount of detail even with such a complex input.

### 4.1. Eye Selection Improvements

The main focus on the eye selection algorithm is to solve the problem of the pupil reflection check, which is tricked by some specific glasses with black frames. This can be done by a replacement with white pixels of the continuous black horizontal lines which may cut the eye selection search area. This way the reflection on the glasses frame will not be detected anymore. This idea still needs implementation and testing.

There is also an optimisation step regarding the moment when the eye ratio map score is computed. The idea is to move this step to after the pupil check is done in order to compute this score on a smaller number of frames. The reason it is not implemented this way at the moment is strictly related to some memory allocations which need to be changed in the software architecture.

### 4.2. Nostrils and Mouth Selection

Nostril selection will be based on the eye location bounding boxes’ output. The search will be performed in the area under the eyes and will have the purpose of containing inside its output bounding box both black areas representing the nostrils. All the procedures of converting the grayscale input picture to black and white should remain similar but the rules and properties for selection will be very different. The nostrils’ output will not have a fixed size for the bounding box.

The mouth selection algorithm will be based on the nostril selection bounding box. The search will be performed in the area under the the nostrils and will have the purpose of containing the whole mouth inside its output bounding box. This part may be more difficult because the color of the mouth in the black–graywhite converted picture differs between different subjects and this will require some special branching for a different color search.

In the future, the output algorithm will be represented by these four bounding boxes, and the neural network trained using the generated dataset will have a similar output.

## 5. Conclusions

This paper introduced an efficient and functional algorithm for eyes detection on very specific driver recordings generated using an automotive infrared sensor. The algorithm was especially designed to be very accurate but not necessarily constant. It is not meant to be used in real time scenarios but in the future it may be run for demonstration reasons against the neural network, which will be trained using its output. Its processing speed allows it to be used in real time.

Datasets created using this method are already used for training neural networks and the first results are very promising. This demonstrates that the purpose of generating ground truth data for eyes location using this algorithm has already been achieved.

## Data Availability

Data are available in the internal storage of Continental Automotive Romania.
